# Machine learning to predict pregnancy outcomes: a systematic review, synthesizing framework and future research agenda

**DOI:** 10.1186/s12884-022-04594-2

**Published:** 2022-04-22

**Authors:** Muhammad Nazrul Islam, Sumaiya Nuha Mustafina, Tahasin Mahmud, Nafiz Imtiaz Khan

**Affiliations:** Department of Computer Science and Engineering, Military Institute of Science and Technology, Dhaka, 1216 Bangladesh

**Keywords:** Machine learning, Literature review, Pregnancy, Childbirth, Data science, Neural network, Supervised learning

## Abstract

Machine Learning (ML) has been widely used in predicting the mode of childbirth and assessing the potential maternal risks during pregnancy. The primary aim of this review study is to explore current research and development perspectives that utilizes the ML techniques to predict the optimal mode of childbirth and to detect various complications during childbirth. A total of 26 articles (published between 2000 and 2020) from an initial set of 241 articles were selected and reviewed following a Systematic Literature Review (SLR) approach. As outcomes, this review study highlighted the objectives or focuses of the recent studies conducted on pregnancy outcomes using ML; explored the adopted ML algorithms along with their performances; and provided a synthesized view of features used, types of features, data sources and its characteristics. Besides, the review investigated and depicted how the objectives of the prior studies have changed with time being; and the association among the objectives of the studies, uses of algorithms, and the features. The study also delineated future research opportunities to facilitate the existing initiatives for reducing maternal complacent and mortality rates, such as: utilizing unsupervised and deep learning algorithms for prediction, revealing the unknown reasons of maternal complications, developing usable and useful ML-based clinical decision support systems to be used by the expecting mothers and health professionals, enhancing dataset and its accessibility, and exploring the potentiality of surgical robotic tools. Finally, the findings of this review study contributed to the development of a conceptual framework for advancing the ML-based maternal healthcare system. All together, this review will provide a state-of-the-art paradigm of ML-based maternal healthcare that will aid in clinical decision-making, anticipating pregnancy problems and delivery mode, and medical diagnosis and treatment.

**Supplementary Information:**

The online version contains supplementary material available at (10.1186/s12884-022-04594-2).

## Introduction

According to WHO, 810 women die every day in this world due to childbirth and pregnancy-related complications, while the majority (94%) of all maternal deaths occur in low and lower-middle-income countries [1]. Due to the recent advancement in technology, the rate of maternal deaths is reducing [2, 3], yet is a challenging task to ensure the safety of both mother and child during pregnancy. In such a scenario, the pregnancy-related risks can be reduced by forecasting the complications and by taking preventive measures. Thus, the use of predictive modeling became emergent to save the lives of millions of mothers and infants.

Obstetric complications such as preeclampsia, prolonged labor, and the like are the primary reasons for such deaths [4]. Unfavorable delivery circumstances, such as severe blood loss, failure to progress (FTP) labor, abnormal presentation of fetus, preterm birth, and others, can result in severe maternal complications [5], putting both the mother’s and the baby’s lives at risk. However, most of these complications are avoidable and suitable measures can be taken to ensure a risk less delivery procedure. For example, in case of abnormal position and presentation of the fetus, cesarean section or forceps delivery could be the safer delivery procedure [6, 7].

In recent years, some studies have been carried out to predict certain risks that can occur during pregnancy and predict the birth method suitable to the pregnancy characteristics of mothers. For example, Pereira et al. [8] predicted the most suitable delivery method among vaginal, cesarean, forceps, and vacuum delivery using different supervised machine learning (ML) algorithms. In another study, Chen et al. [9] predicted the factors associated with preterm birth using a Neural Network (NN) and Decision Tree (DT) algorithm. Similarly, Rawashdeh et al. [10] predicted the risk of premature birth using Random Forest (RF), DT, K Nearest Neighbors (KNN), and NN. Different ML techniques were used in these studies showing varying performances. Again, the type of data used in these studies was also different, that includes, for example, demographic factors, maternal factors, obstetric characteristics, medical and obstetric history, ultrasound measurements, behavioral parameters, and suchlike.

The primary objective of this study is to explore the state-of-the-art views of research and development focusing on ML to forecast and detect different conditions of pregnancy. The objective can be achieved through the following secondary objectives; to examine scopes and publication profiles of the existing studies(RO1); to explore the data types used for predicting pregnancy outcome (e.g., type of childbirth method, suitability of vaginal birth, vaginal birth after cesarean section and the likes) (RO2); to examine the use of different ML algorithms for predicting mode of childbirth, complications during childbirth, etc. (RO3); and to find out the gaps in the existing literature and recommend future research opportunities (RO4). To attain these objectives a systematic literature review (SLR) approach [11] is adopted.

The rest of this article is organized as follows: a theoretical background for this review study is described in “Theoretical background” section. “Study methodology” section briefly discusses the methodology followed throughout the study. “Analysis of extracted data” section analyzes selected articles in terms of correlation among reviewed studies, publication year and article type, study objectives, type of features and algorithms used, the performance of ML algorithms, and the context (country) of these studies. “Study findings” section summarizes the findings from reviewed articles. Future research opportunities in relevant areas of pregnancy complications along with an ML healthcare framework are discussed in “Future research implication” and “Future research framework” sections, respectively. Finally, a conclusion with the limitations of this study is presented in “Future research framework” section followed by references and appendix.

## Theoretical background

This section discusses machine learning techniques and pregnancy complications.

### Machine learning

ML is a subset of artificial intelligence, which is one of the most rapidly growing technical fields [12]. With the vast expansion of structured and unstructured data, also known as big data, ML has become indispensable as it’s unfeasible to handle this data with various methods [13]. Big data enables ML algorithms to uncover unknown patterns which stimulate the process of decision-making. Machine learning is the field in which machines are taught to resemble human behavior. It emphasizes the use of data and algorithms. Handling a large volume of data, training, and building a machine learning model, as well as training that model to gain improved accuracy, are all part of the ML technique. The learning of machines or models in ML depends on the human intervention on raw data. The data can be labeled or unlabeled. Based on this data the ML model estimates a pattern about the data. Then it uses a function where the estimation is compared to the known answer i.e. the labeled data, to determine accuracy. The model then tries to fit the estimation to the known data points so that accuracy can be further improved. This is how the ML technique trains and builds up the models that help the machine imitate human behavior.

In today’s world, machine learning is employed extensively in a variety of fields. ML techniques are primarily used for classification and prediction (e.g., medical diagnosis, forecasting pandemic [14]), clustering analysis (e.g., securing the web by detecting unusual traffic, identification of cancer cells, partitioning customers), natural language processing (e.g., speech recognition, language translation, sentiment analysis), and the list goes on. ML is being used broadly in the healthcare sector to process electronic health records (EHR) and to implement clinical decision support tools using EHR. EHR along with ML can be used in disease identification and diagnosis (kidney disease prediction [15], breast cancer prediction [16] and diabetes prediction [17]); predicting cesarean childbirth[18], finding the best features for predicting the modes of childbirth [19]; detecting an unknown pattern in the medical records, producing medicine by analyzing genome data, and the likes. With the aid of ML techniques, it’s possible to predict different complications that can occur in pregnancy in advance of child delivery.

### Types of machine learning

**Fig. 1:**
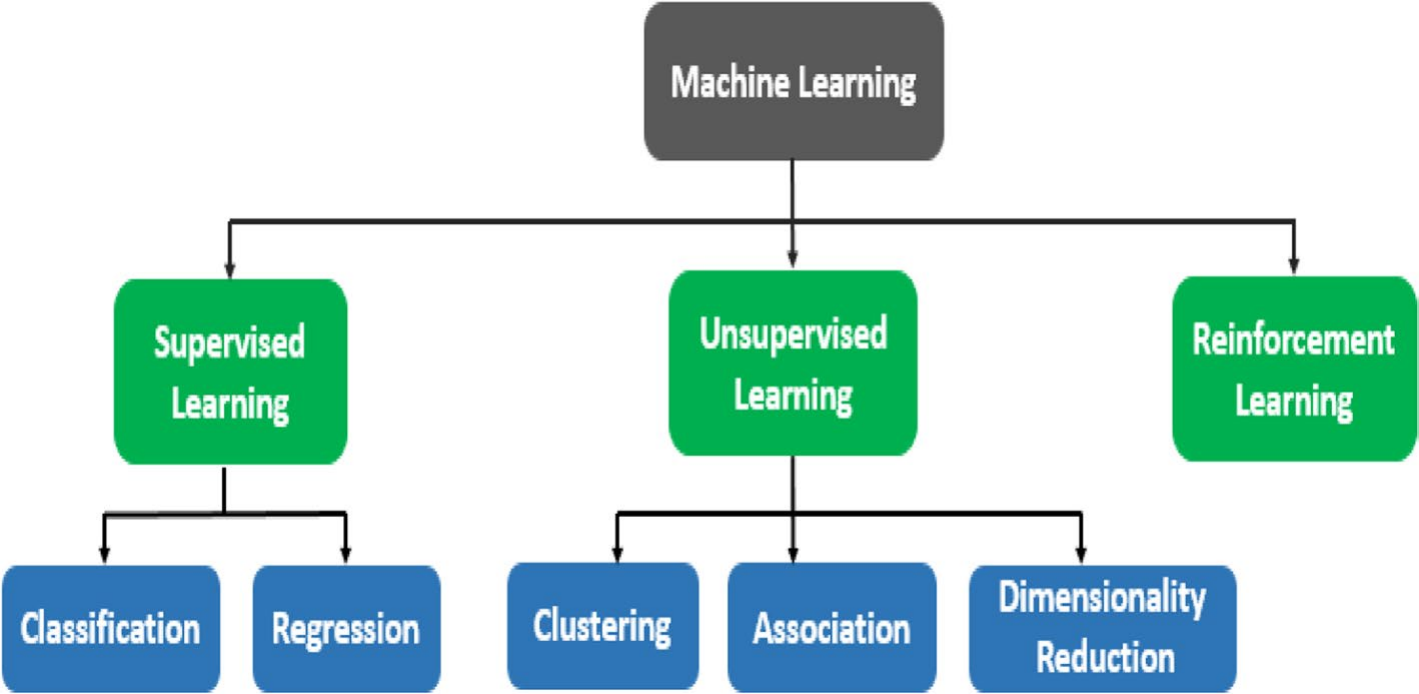
Different types of machine learning
- Unsupervised Learning
- Reinforcement Learning

Machine learning (ML) can be categorized in many ways. Though there are three largely recognized categories of machine learning depending on how the system (ML model or agent) is trained, which are shown in Fig. 1:

- Supervised Learning

This section briefly discusses the types of machine learning and some of its real-world applications.

- *Supervised Learning:* In supervised learning, the datasets are labeled to train algorithms to classify data or predict outcomes [20]. Supervised learning can be further categorized into regression and classification tasks. Classification algorithms predict the class label of new data points (test data) depending on how the model is supervised by training data [21]. For example, Gynecologists can predict the mode of childbirth by using classification algorithms [19]. For this, ML model has to be trained by actual childbirth records, containing different birth modes and features. The features are the input of the model, while the ‘mode of childbirth’ is the output of the model. The model identifies a relation between the input features and the output feature, and later on, the relation is used to predict the mode of childbirth for new/unknown instances.
  *Regression* algorithms identify correlations between dependant and independent variables to predict the continuous value of the dependant variables. For example, predicting height of a child, based on father’s and mother’s height. In this scenario, father’s and mother’s height are independent variables, while child’s height is dependent variable. The model identifies a relation between the independent and dependent variables, later on, by using that relation, the model can predict the height of a child, whose data was not present in the training dataset.
- *Unsupervised Learning:* Models are not supervised using training datasets in the unsupervised learning approach [22]. Instead, models learn from the hidden pattern and unknown information from the datasets. There are three types of unsupervised learning that include, clustering, association, and dimensionality reduction.
  *Clustering* technique groups data points together based on their similarities. Dissimilar objects are grouped in distinct groups, whereas similar objects remain in the same group [23]. For detecting brain tumor from MRI images, clustering algorithms can be utilized [24]. These algorithms can group images into two clusters, one delineating the group of images containing brain tumor, while the other portraying the group of images containing no brain tumor.
  *Association* is an unsupervised learning approach that uses rules to discover relationships between features in a dataset.
  To efficiently utilize the vast amount of structured and unstructured data with optimum usage of resources it might be necessary to reduce the volume of data. Some of the input features might not play any role in the ML model’s performance and furthermore, data might come with redundant noises. *Dimensionality reduction* is used to adequately handle the complex and massive data so that less computing resources and storage will be used by other ML algorithms. [25].
- *Reinforcement Learning: Reinforcement learning* is an area of ML concerned with how the agents can take actions in an environment that will result in a maximum reward [26]. An agent is an entity making decisions based on rewards and punishments. The way reinforcement learning differs from supervised learning is, instead of learning from a dataset a reinforcement learning agent learns from the consequences of its actions.

### Maternal complications

The common maternal complications responsible for the majority of maternal deaths are gestational diabetes, severe bleeding, infection, preeclampsia, eclampsia, prolonged labor, preterm labor, and unsafe abortion [27]. These issues are briefly presented below.

*Gestational Diabetes Mellitus (GDM):* GDM (gestational diabetes mellitus) is a situation that may arise during pregnancy, in which the placenta produces a hormone that hinders the body from adequately utilizing insulin. Instead of being absorbed by the cells, glucose builds up in the blood and causes blood sugar levels to rise [28]. This may cause premature birth, preeclampsia, overweight babies (Macrosomia), and low blood sugar in babies after delivery (Hypoglycemia).

*Infection:* Infections can cause complications for the pregnant mother and her baby during pregnancy. Infections may lead to miscarriage, preterm labor, and birth defects in infants.

*Preeclampsia:* Preeclampsia is a medical condition that occurs after 20 weeks of pregnancy and causes high blood pressure and kidney problems. Women who are reported with vision problems and swelling of the legs, body or face can be considered as having symptoms suggestive of preeclampsia [29]. There are also other symptoms of preeclampsia such as proteinuria (excess protein in urine), headaches, impaired liver function, etc.

*Eclampsia:* Eclampsia is an unusual pregnancy condition that causes seizures as a consequence of severe preeclampsia. Permanent neurological damage from recurring seizures or intracranial bleeding, renal insufficiency and acute renal failure, and other complications might arise as a result of eclampsia [30].

*Preterm labor:* The presence of uterine contractions of sufficient frequency and amplitude to cause progressive effacement and dilation of the cervix prior to term gestation is characterized as preterm labor [30]. In simple words, when labor begins before 37 weeks of pregnancy, it is referred to as preterm labor. Preterm labor can result in premature birth. Respiratory distress, heart problems, impaired learning, cerebral palsy, and hearing loss are some short and long-term medical issues for premature newborns.

These issues can physically and mentally distress the mother and/or baby, resulting in mild to life-threatening situations. Some of these problems can occur due to past medical conditions such as diabetes, family history of preeclampsia, previous cesarean delivery, previous surgery and due to bad habits such as smoking, drinking alcohol [31].

## Study methodology

A systematic review can be considered as a means to summarize, evaluate and analyze the existing studies of a particular research topic or area. To attain the review objectives, a systematic literature review (SLR) approach provided by Kitchenham et al. [11] was followed.

### Search strategy

The primary articles or studies were searched in different sources such as Google Scholar, SpringerLink, IEEE Xplore, ScienceDirect, etc. The articles were searched using some keywords and their synonyms, for example, the keywords that were used are ‘pregnancy outcome prediction and machine learning,‘pregnancy risk prediction and machine learning, ‘vaginal birth prediction and machine learning’, ‘cesarean birth prediction and machine learning’, ‘preterm birth prediction and machine learning’, ‘risk and pregnancy determine and machine learning’, ‘vaginal(normal) delivery after c section or cesarean section and machine learning’, ‘cesarean section or c section prediction and machine learning’, ‘mode of childbirth prediction and machine learning’, ‘premature birth prediction and machine learning’, ‘risks prediction during pregnancy and machine learning’. Later ‘machine learning’ was replaced by related keywords like data mining, artificial intelligence, and deep learning in the searching procedure. The studies published in the last 21 years(2000-2020) were included in the review. Different types of publications e.g., journals, conference articles, open research articles were searched for finding the maximum number of related articles.

### Inclusion and exclusion

In order to ensure that only suitable articles are being selected for this study, some eligibility criteria were considered. Out of the 241 studies, only 26 studies were considered for the systematic review. The few selected articles were chosen using some inclusion-exclusion criteria. A study was eligible for reviewing if it met all the following criteria: (a) studies related to predicting pregnancy outcome, mode of delivery, and pregnancy complication adopting ML techniques; (b) full-text research articles; and (c) studies published in between 2000 to 2020.

The review had the following exclusion criteria: (a) abstract only studies; (b) duplicate articles; (c) articles written in languages other than English; (d) reported outcomes inconsistent with objective; and (e) solely theoretical works. If an article undeniably met one or more of these criteria, it was ruled out from later review.

**Fig. 2:**
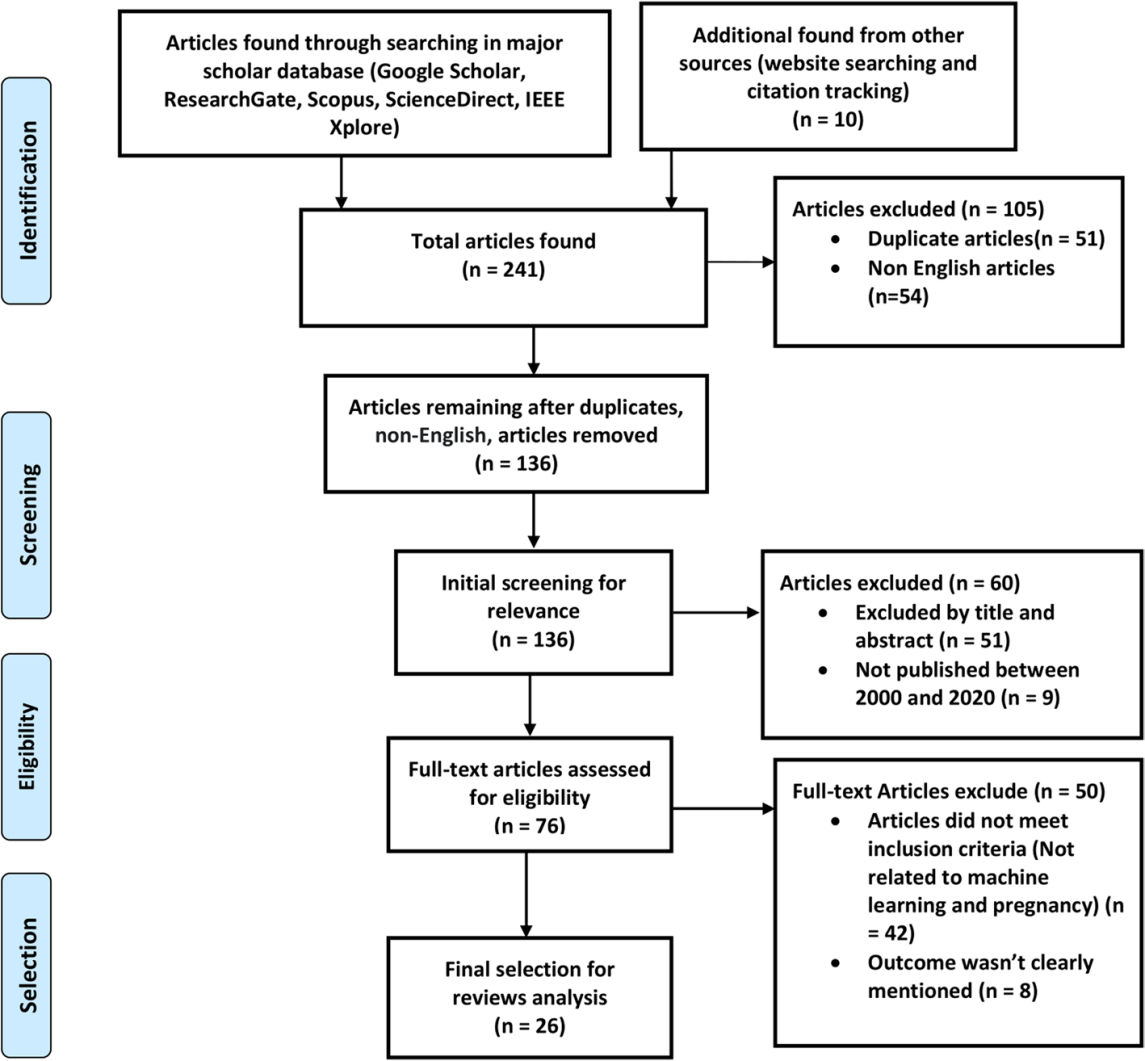
PRISMA flow diagram for the selection of articles

The summary of the search and selection of final articles are illustrated in Fig. 2. The papers were selected by focusing on the abstract and introduction mainly. 241 research works were discovered as primary materials during the preliminary search. 136 articles were chosen after duplicates, non-English articles were removed. After evaluating articles’ titles and abstracts, the first level of screening yielded 76 articles excluding 60 articles. Following that, after reading the abstract and introduction, and methodology, the next level of screening was carried out, yielding a list of 26 articles that were selected for the final review analysis.

### Data extraction and analysis

**Fig. 3:**
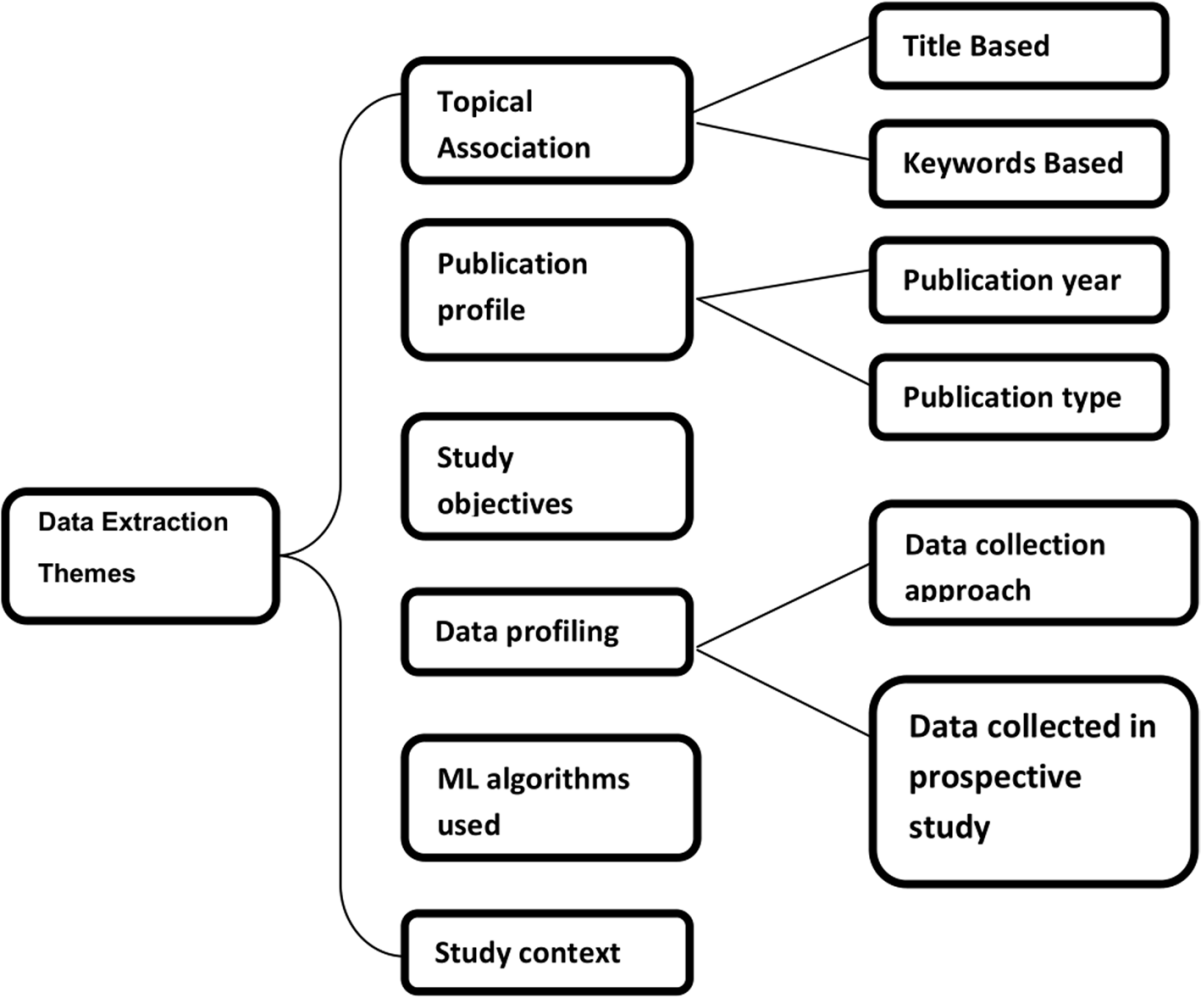
Themes for data extraction

While extracting core characteristics and pertinent information from the selected primary studies in a systematic manner, six prominent themes were considered as shown in Fig. 3. The types of data obtained from the reviewed articles can be easily perceived with the help of these six themes. The themes are briefly discussed below to outline the extracted data in a comprehensive manner.

- *Topical Association:*In this theme, the association and relevance among the reviewed articles were investigated by assessing the word clouds generated considering articles’ titles and keywords.
- *Publication Profile:* This theme depicts the publication year of the reviewed articles and the types of the papers like journal papers, conference papers, etc.
- *Study objectives:* This theme points out the key objectives of the reviewed articles. The study objectives were grouped into similar scopes, and mapping was drawn up based on publication year.
- *Data profiling:* This scheme classifies the reviewed articles based on the data collection approach and type of features used.
- *ML algorithms used:* The algorithms used in different papers were pointed out in this theme. The frequency and performance of these algorithms were analyzed. Moreover, mapping was carried out among study objectives, feature types, and algorithms.
- *Study context:* The research works were carried out in different geographical locations. The scope and features considered from a specific region were also investigated.

The extracted data was properly organized to serve the aim of evaluating, analyzing, and summarizing the existing research.

## Analysis of extracted data

### Topical association

**Fig. 4:**
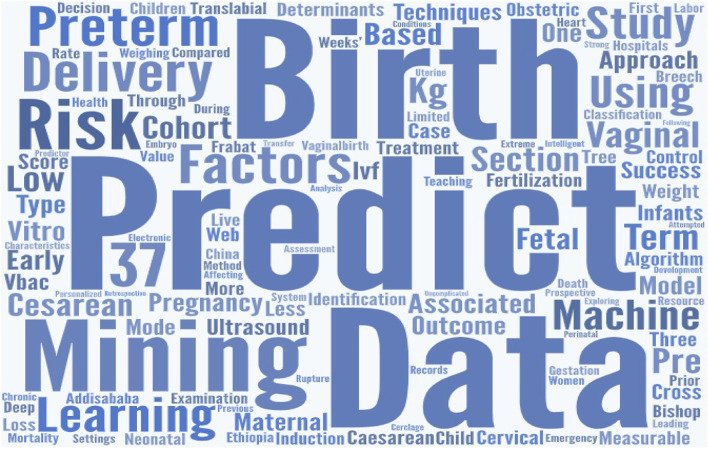
Word cloud based on the title of the articles

**Fig. 5:**
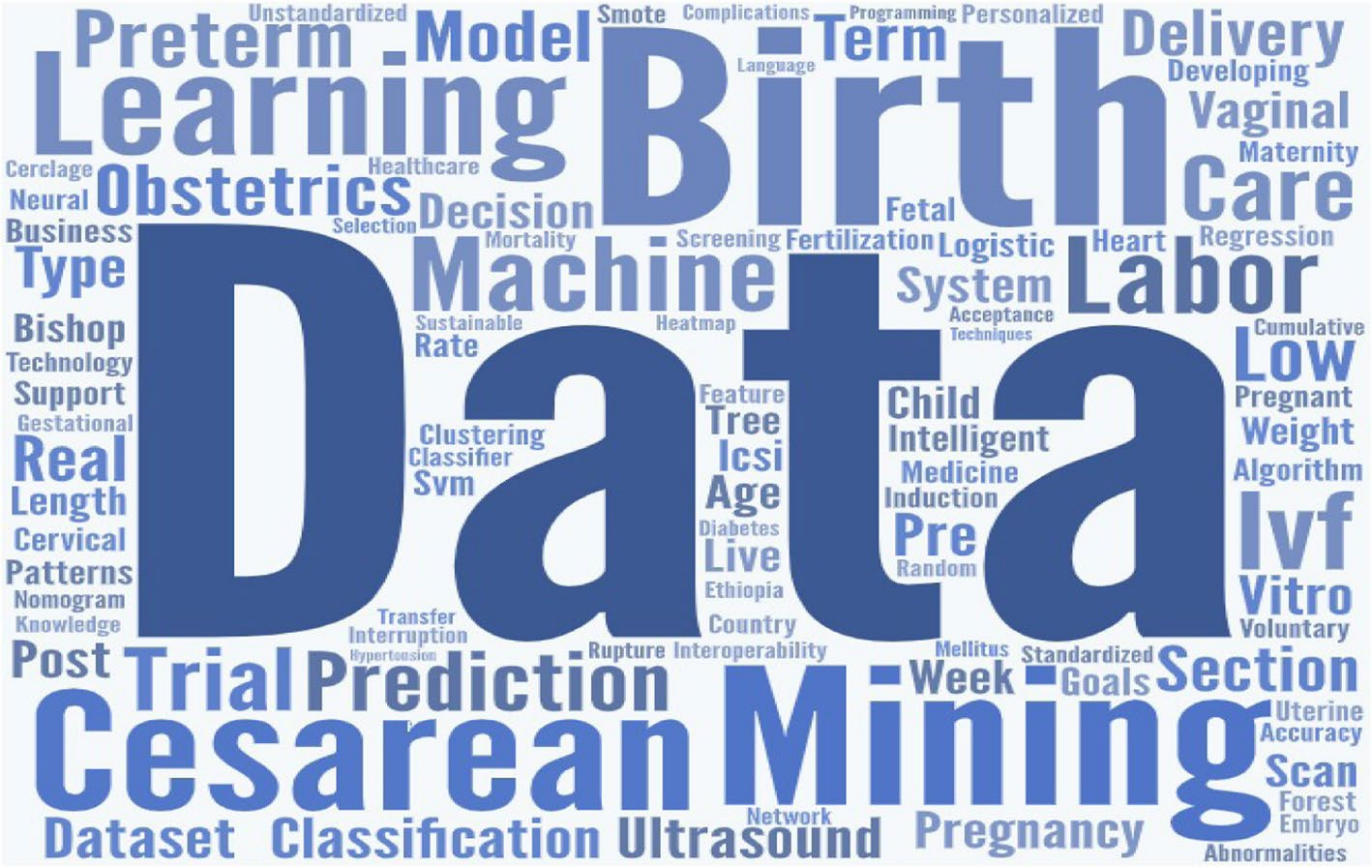
Word cloud based on the keywords of the articles

Word cloud is an effective and stunning visualization method for text analysis [32]. It is an intuitive way of portraying what kind of topics are covered in the text body without providing too many details. Word clouds can be used to verify the relationship between the articles under evaluation. Two-word clouds were generated, one is generated based on the article titles (see Fig. 4) and another one is based on the keywords of the reviewed articles(see Fig. 5). Figure 4 indicates the terms appeared more often in the study titles. For example, predict, birth, data, mining, delivery, machine, learning, factors, risk, and preterm are the most highlighted words. Again, Fig. 5 shows the frequent keywords in the selected papers. As such data, mining, birth, machine, learning, cesarean, prediction, preterm, labor, and care are the highly focused words. The analysis showed birth, data, mining, machine, learning, and preterm appeared frequently in both considerations. In the word cloud, the bigger the words are the ones that are often mentioned and the font size indicates the categories/topics discussed in the text body. These results thus indicated that the selected articles are closely associated and focused on machine learning, data mining, and predicting various aspects of pregnancy.

### Publication profile

**Fig. 6:**
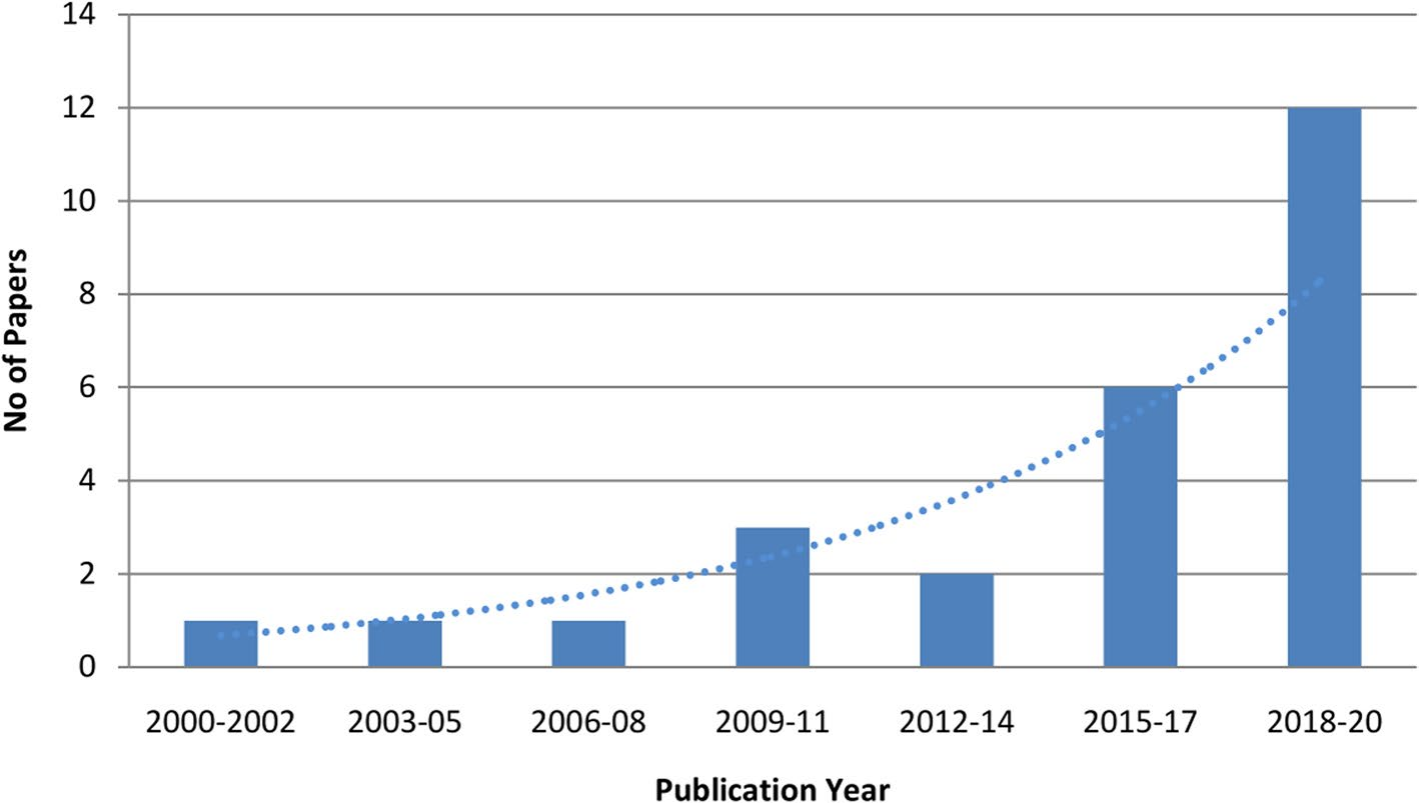
Publication trend since 2002

The research articles that were published every three years ranging from 2000 to 2020 are presented in Fig. 6. It was revealed that the related papers were published in the years 2002, 2003, 2008, 2010, 2011, and 2013-2020 whereas no related papers were published in the following years: 2000-2001, 2004-2007, 2009, and 2012. According to the trend, there has been a resurgence of interest in research conducted towards maternal welfare with machine learning since 2015. Moreover, the slope of the trend-line in Fig. 6 is exponential as the number of articles raised rapidly. During 2015-19, the number of articles grew slowly, but during 2018-20, the articles climbed dramatically and peaked in 2020.

Among the 26 selected papers, twenty of them were journal papers and six of them were conference papers. Among the journals six were published by Elsevier, four were by Springer, two by BMJ and the following publishers published one journal each, BioMed Central, PLOS, SciTePress, Karger Publishers, Wiley Online Library, Mapana Journal of Sciences, JBRMS, and American Medical Association. Some of the academic journals in which the articles were published are presented in Table 1. Most of the journals primarily focused on computer science or machine learning, pregnancy, and healthcare. Hence, inspecting publication trends (Fig. 6) and journal lists (Table 1), it was indicated that there is an emergence of health informatics studies focusing on pregnancy and machine learning.

**Table 1:** Primary focus of the academic journals where reviewed articles have been published

Name of the academic journal	Reviewed Article	Focus
Procedia Computer Science	[8, 33]	Information Systems, health informatics
BMC Pregnancy and Childbirth	[34, 35]	Pregnancy and childbirth
American Journal of Obstetrics and Gynecology	[36, 37]	Obstetrics and gynecology
Gynecologic and Obstetric Investigation	[38]	Obstetrics and gynecology
Journal of Investigative Medicine	[39]	Medical research
Ultrasound in Obstetrics and Gynecology	[40]	Medical research
BMJ Open	[41]	Medical research
Neural Computing and Applications	[42]	Neural computing
PloS one	[43]	Science, engineering and medicine
Computer Methods and Programs in Biomedicine	[44, 45]	Biomedical informatics, medical research
Journal of Basic Research in Medical Sciences	[46]	Medical research

### Study objectives

In this section, the reviewed articles were disseminated into six scopes after examining the objectives. Table 2 summarizes and classifies the selected studies in terms of such six categories.

**Table 2:** Objectives of the reviewed articles

Scope	Study Objective	Ref	Frequency
Predicting pregnancy risks/complications	Predicting risk level during pregnancy	[33]	
	Explore risks related to voluntary termination of pregnancy	[47]	
	Prediction of preterm/extreme preterm birth	[10, 48–51]	9 (35%)
	Prediction of risk of uterine rupture	[37]	
	Prediction of risk of perinatal death	[52]	
Exploring pregnancy factors	Determining factors related to successful vaginal delivery	[35]	
	To explore factors responsible for emergency cesarean section	[39]	
	To Determine influential factors in child mortality prediction	[44]	7 (27%)
	To explore factors responsible for preterm birth	[9, 53]	
	Prediction of low birth weight and factors responsible for it	[46, 54]	
Predicting mode of delivery	To predict delivery method	[8]	4 (15%)
	To predict success of vaginal birth after cesarean delivery	[36, 38, 41]	
Predicting outcome of IVF treatment	Predicting early pregnancy loss	[45]	
	Predicting successful pregnancy after IVF	[42]	3 (11%)
	Predicting the live birth chance	[55]	
Predicting labor outcome	To determine the suitability of induction of labor	[34]	2 (8%)
	To determine potential value of cervical length in predicting progress of labor	[40]	
Comparison between two birth weight groups	To compare outcome of vaginal intended breech deliveries between low weight group and high weight group	[43]	1 (4%)

The majority (35%, *n* = 9) of the studies were concerned about predicting risks/ complications in different pregnancy circumstances. These risks/ complications included preterm birth, maternal death, and uterine rupture. Under the scope of predicting pregnancy risks/ complications, five studies with the primary objective of predicting preterm pregnancy were found. For instance, Rawashdeh et al. [10] developed a decision support system for predicting premature delivery where two decision classifications were used, one for indication of premature birth and another for timed pregnancy. Similarly, Gaoa et al. [49] focused on predicting extreme preterm birth with electronic health records. Positive and negative classifications were used to specify the state of preterm birth.

Next, some studies (27%, *n* = 7) were dedicated to exploring/determining pregnancy factors for certain scenarios. Birara et al. [35] explored factors associated with successful vaginal delivery. Similarly, Guan et al. [39] analyzed 22 maternal and fetal factors which might be responsible for emergency cesarean section, while Chen et al. [9] and Rawashdeh et al. [10] identified the influential factors for preterm birth.

Some other studies (15%, *n* = 4) were focused on predicting the mode of delivery. For example, Pereira et al. [8] used obstetric risk factors to predict suitable mode of delivery among four classes (vaginal, cesarean, forceps, vacuum). Similarly, in other three studies, [36, 38, 41] the objective was to predict the success of vaginal birth after cesarean section. Li et al. [36] presented two decision classifications to determine the mode of delivery (vaginal and unplanned cesarean delivery).

The outcomes of pregnancy following IVF treatment were evaluated in three studies. Oiu et al. [55] predicted live birth chance in terms of percentage, prior to first IVF treatment. Similarly, Hassan et al. [42] predicted the outcome of pregnancy after IVF where two classes (successful, unsuccessful) specified the outcomes. Again, Liu et al. [45] predicted early pregnancy loss after in vitro fertilization-embryo transfer.

Two studies focused on determining the outcome of labor. Khazardoost et al. [34] investigated, between Bishop Score and transtibial ultrasound measurements are more applicable in determining the suitability of induction of labor. Similarly, Ramanathan et al. [40] analyzed if the measurement of cervical length can determine labor outcome.

Finally, in one study Jennewein et al. [43] compared the maternal and neonatal outcomes of vaginal intended breech deliveries between two birth weight groups (birth weight 2.5 kg – 3.79 kg and birth weight ≥ 3.8 kg).

The research objectives of the examined papers were mapped by published years to explore the research trend from 2000 to 2020. Six of the nine studies on pregnancy complications prediction were published between 2015 and 2020. Between 2015 and 2020, four research on exploring pregnancy characteristics were conducted. In 2019-2020, most of the published articles were (11%, *n* = 3) concentrating on predicting IVF treatment outcomes, while two studies focused on predicting mode of delivery. Thus, the findings indicated that, interest in studies predicting mode of delivery and IVF treatment outcomes increased very recently.

### Data profiling

The datasets used in the reviewed studies were examined in terms of data collecting procedures and the types of features included. Table A1 provides an overview of the types of data and features used in the selected articles. Table A2 and Table A3 give an overview of how the features are categorized and the accuracy of different algorithms in different studies respectively.

All data were of electronic health records (EHR) and collected directly from a clinic, medical institute, and from the study-participants in cohort studies. An EHR is a database that stores information about a person’s health and is collected during one or more visits to any healthcare facility [56]. Data collection procedures were assigned into four categories; independently collected, prospective cohort study, retrospective cohort study, and case-control study.

(i) *Independently collected:* In the majority of studies (*n* = 14), data was collected by the researcher from clinical institutions. For example, Hassan et al. [42] collected infertility data of 1729 patients from a clinic in Istanbul, Turkey; while Pereira et al. [8] used the ERH data from Centro Hospitalar of Oporto, Portugal during the period of 2012-2015.
(ii) *Prospective cohort study:* In some studies (*n* = 4), data were collected through a prospective or retrospective cohort study. In cohort studies, end results of one or more groups exposed to *outcome of interest, disease or factors* are compared with the end result of the group who are not exposed to the outcome of interest, disease, or factors [57]. A prospective cohort study measures the exposures of interest, factor, or disease on study participants [58]. All of the data is collected prospectively in this sort of study design. For example, in Chen et al. [9] the dataset was collected prospectively where gestation week of the pregnant women was 26 or more. Similarly, in a prospective cohort study [43], data was collected from 1,054 patients who intended vaginal breech delivery.
(iii) *Retrospective cohort study:* A retrospective cohort study, also known as a historical cohort, is devised after people have already developed the outcomes of interest [58]. In many (*n* = 7) reviewed studies data was collected retrospectively. For example, in the historical cohort [38] study, 599 sample records of pregnant women who attempted trial of labor after cesarean, was collected from 2000 to 2010 in Mayo Clinic (USA).
(iv) *Case-control study:* In the single case-control study, Birara, and Gebrehiwot [35], 204 sample records were used. The case group included 101 samples of vaginal delivery and 103 samples of vaginal birth after one cesarean section were in the control group.

Again, a total of eleven types of features were found in the dataset used in the reviewed studies (see Table A1). *Demographic factors* refers to socio-economic characteristics of mothers, e.g., age, education, ethnicity, religion, marital status, and suchlike. *Maternal factors* includes physical traits of the mother (e.g., height, weight, body mass index (BMI)). *Obstetric characteristics* relates to pregnancy factors, childbirth factors, and complications that can arise in pregnancy. For example, mode of delivery, sex of the baby, birth weight, gastrointestinal disease, gestational diabetes, and suchlike. *Medical and obstetric history* includes previous medical obstetric records such as parity, previous vaginal delivery, history of gestational diabetes, previous miscarriage, previous cesarean delivery. *Medical history of relatives* involves obstetric issues of relatives like mother/sister with preeclampsia, family history of gestational diabetes. *Current medical record* is the current health information, health issues, disease record of mothers (i.e., blood pressure, weight gain, number of physician visits, glucose level). *Pregnancy termination attributes* is the features incorporated in pregnancy termination. *Behavioral parameters* are the attributes related to mothers’ lifestyle and addiction (e.g., smoking, drug and alcohol). *Infertility characteristics* are the features taken into consideration in the process of IVF treatment (e.g., type of infertility, duration of infertility, infertility diagnosis, and suchlike).

It was noticed that demographic factors, maternal factors, and obstetric characteristics were the most common types of features used in all studies; while infertility characteristics were used in studies aimed at predicting the outcome of IVF treatment; and ultrasound characteristics were used in several studies aimed at predicting childbirth mode.

### ML algorithms used

The reviewed studies found that different ML algorithms were used in different studies for specific purposes. For example, Li et al. [41] used Multivariate Analysis (MA) and Univariate Analysis (UA) for predicting the success of vaginal birth after cesarean delivery, while for the same purpose Lipschuetz et al. [36] used Random Forest (RF), AdaBoost Ensemble (AE), and Gradient Boosting (GB).

Table 3 elucidates the algorithm used in different studies. The studies showed that Support Vector Machine (SVM) was used in the maximum number of studies (*n* = 9) while Balanced Random Forest (BRF), GB, AE, Recurrent Neural Network (RNN), Back Propagation Neural Network (BPNN), Classification And Regression Trees (CART), Multilayer Perceptron Neural Networks (MLP), PART, Clustering PAM and K Means Cluster (KMC) analysis were used in single studies. After SVM, MA (*n* = 8), RF (*n* = 8), DT (*n* = 7), UA (*n* = 5) and NB (*n* = 4) were used in most of the studies. Apart from that, other machine learning algorithms like the Generalized Linear Model (GLM), J48, Logistic Regression (LR), etc. were used in different studies to get desired outcomes. Table 3 shows the algorithms and the reference of the studies where the algorithms were used.

**Table 3:** Algorithms used in different studies

Algorithm	Reference
Decision Tree (DT)	[8, 10, 45–47, 54, 54]
Logistic Regression (LR)	[37, 45, 51, 52, 54, 55]
Generalized Linear Model (GLM)	[8, 47]
K Means Cluster (KMC)	[48]
Support Vector Machine (SVM)	[8, 42, 45, 47, 50, 51, 53–55]
J48	[44, 46]
Naïve Bayes (NB)	[8, 46, 53, 54]
PART	[44]
Multivariate Analysis (MA)	[34, 35, 38–41, 43, 52]
C5.0 Decision Tree (DT)	[9]
Random Forest (RF)	[10, 36, 42, 45, 46, 50, 54, 55]
XgBoost (XB)	[45, 55]
Balanced Random Forest (BRF), AdaBoost Ensemble (AE), Gradient Boosting (GB)	[36]
K Nearest Neighbors (KNN)	[10, 50]
C4.5 Decision Tree (DT)	[33, 42]
Clustering PAM	[53]
Univariate Analysis (UA)	[38–41, 52]
Random Tree (RT), Decision Table	[46]
Neural Network (NN)	[9, 10, 54]
Recurrent Neural Network	[49]
Back Propagation Neural Network (BPNN)	[45]
Classification And Regression Trees (CART)	[42]
Multilayer Perceptron Neural Networks (MLP)	[42]

The performance of the algorithms was analyzed and found that different algorithms showed different results in terms of accuracy, sensitivity, specificity, etc.(see Table A3). For example, Pereria et al. [8] achieved 83.91% accuracy for predicting the type of delivery by identifying obstetric risk factors using DT, while Ghaderighahfarokhi et al. [46] got 95% accuracy for predicting low birth weight infants and associated factors using DT. Again under the same scope or category, different algorithms gave the best accuracy in different studies. For example, to predict complications in pregnancy Senthilkumar et al. [54] gained the highest accuracy(89.95%) using DT, Tesfaye et al. [44] got the best accuracy (94.3%) using the J48 algorithm; Ghahfarokhi et al. [46] got 98% accuracy using the Random Tree (RT) and J48; Malea et al. [53] gained 88% accuracy as the best result using NB.

The association among algorithms, features, and study objectives of the selected articles is presented in Table 4. It shows that different algorithms were used for different study purposes. It also shows the different features categories on which the chosen algorithms were implemented. It is evident from this table that supervised machine learning algorithms (DT, SVM, RF, NB, etc.) are mostly used for different study objectives. It is mainly due to the datasets used. This type of algorithm gives the best result in a labeled dataset that makes it easier to train the model. Hence this makes the supervised machine learning algorithms the most suitable for achieving the different study objectives of the selected articles. The frequent types of features used in these studies are demographic factors, obstetric characteristics, maternal factors, medical and obstetric history.

**Table 4:** Association among the study objectives, feature types and algorithms

Study Objectives	Feature Category	Algorithms
To predict delivery method	Demographic factors, obstetric characteristics, maternal factors	DT, NB, SVM, GLM
To compare maternal and neonatal outcome of vaginal intended breech deliveries between low weight group high weight group		MA
To predict success of vaginal birth after cesarean delivery	Demographic factors, obstetric characteristics, maternal factors, medical and obstetric history, neonatal features, ultrasound measurements, behavioral parameters	MA, UA, RF, AE, GB
Prediction of preterm/extreme preterm birth	Demographic factors, obstetric characteristics, maternal factors, current medical record, medical and obstetric history	DT, NN, RF, KNN
Predicting risk level during pregnancy	Demographic factors, obstetric characteristics, maternal factors, medical and obstetric history	C4.5 DT
Explore risks related to voluntary termination of pregnancy	Demographic factors, obstetric characteristics, medical and obstetric history, pregnancy termination attributes	DT, GLM, SVM
Prediction of risk of uterine rupture	Demographic factors, obstetric characteristics, maternal factors, medical and obstetric history	LR
Prediction of risk of perinatal death	Demographic factors, obstetric characteristics, maternal factors, behavioral parameters	LR, MA, UA
To explore factors responsible for preterm birth	Demographic factors, obstetric characteristics, maternal factors, behavioral parameters, medical and obstetric history, current medical record	NB, SVM, NN, C5.0 DT, clustering PAM
Prediction of low birth weight and factors responsible for it		DT, SVM, RF, NB, NN, LR, J48
Determining factors related to successful vaginal delivery	Demographic factors, obstetric characteristics, maternal factors, medical and obstetric history	MA
To explore factors responsible for emergency cesarean section	Demographic factors, obstetric characteristics, maternal factors, medical and obstetric history, neonatal features	MA, UA
Predicting successful pregnancy after IVF	Demographic factors, maternal factors, medical and obstetric history, ultrasound measurements	SVM, C4.5, RF, CART
Predicting early pregnancy loss during IVF treatment		LR, SVM, DT, BPNN, XB, RF
Predicting the live birth chance after IVF treatment		SVM, RF, LR, XB
To determine the suitability of induction of labor	Demographic factors, maternal factors, medical and obstetric history, ultrasound measurements	MA
To determine potential value of cervical length in predicting progress of labor		MA, UA

### Study context

**Fig. 7:**
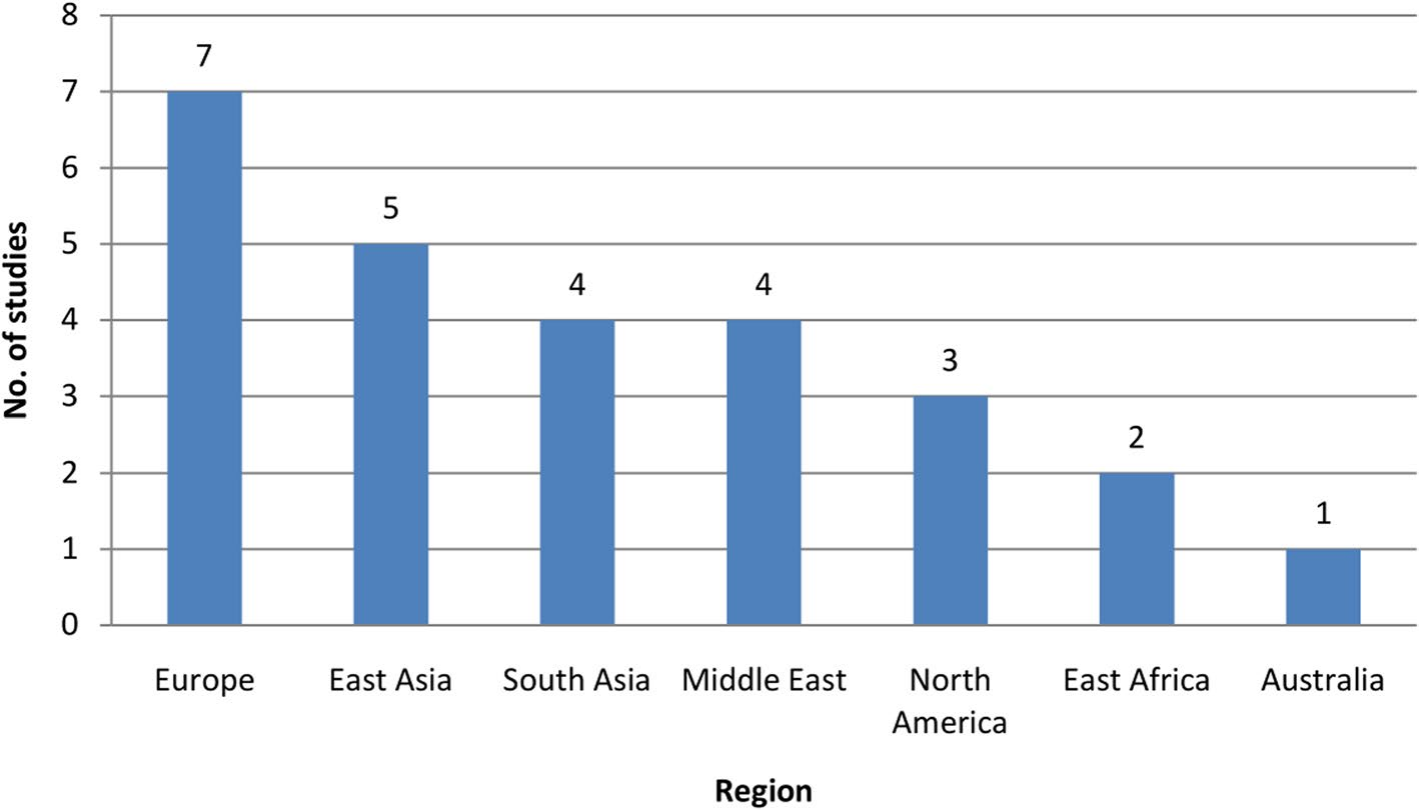
No. of studies in different nations

The existing studies were analyzed in terms of the geographical region where the studies were carried out. The findings showed that most of the studies (*n* = 7) were conducted in *Europe* (see Fig. 7) where two were carried out in *Portugal* and one in each of the following countries: *Germany*, *Romania*,*England*,*Slovenia* and *Scotland*. Three studies (*n* = 3) were carried out in *North America* where all of them were carried out in the *USA*. Few studies (*n* = 2) were conducted in *Africa*. Birara and Gebrehiwot [35] identified factors leading to successful vaginal delivery using demographic and obstetric data of patients collected from three hospitals in Addis Ababa, *Ethiopia*. Some studies (*n* = 9) have been carried out in different parts of *Asia*; four in *India* (South Asia), four in *China* (East Asia) and one in *Taiwan* (East Asia). One study have been carried out in *Australia* and other studies (*n*=4) have been carried out in *Middle East*; two in *Iran*, one in *Israel* and one in *Turkey*.

Again, the review showed that the studies were conducted in some of the developing (*China*, *India* etc.) and developed countries (*USA*, *Turkey*, *Germany* etc.). And the number of researches focusing on maternal safety is very low in most of the developing and underdeveloped countries even though the rate of maternal mortality and morbidity is higher in these regions. For example, out of the 26 selected studies, nine studies were from *South* and *East Asia*. All of these nine studies were carried out in developing countries like *India*, *China* and *Taiwan*. But none of them were from underdeveloped countries or other developing countries of *Asia* like Afghanistan, Myanmar, Nepal.

Table 5 indicates the scopes of the studies and the study context. It shows that the research works done in *North America* are based on the scope of pregnancy risk/ complications and mode of delivery. One among the three studies of the *USA* were concerning predicting preterm birth, while the other two were related to predicting vaginal birth after cesarean delivery [37, 38]. It is not surprising because in recent years there is a trend in vaginal birth after cesarean delivery in the *USA*. The rate of vaginal birth after cesarean delivery increased from 12.4% in 2016 to 13.3% in 2018 [59]. Again, preterm birth complications are quite frequent in the *USA*, preterm birth occurs in approximately 12% of all births in the United States [60]. Also, the types of features used in these studies are related to Demographic factors, maternal factors, obstetric characteristics, medical and obstetric history, and neonatal features.

**Table 5:** Study context, scope and feature types of the reviewed articles

Context		Reference	Scope	Features
North America	USA	[37, 38, 49]	Predicting pregnancy risks/complications, mode of delivery	Demographic factors, maternal factors, obstetric characteristics, medical and obstetric history, neonatal features
Europe	Portugal	[8, 47]	Predicting pregnancy risks/complications, mode of delivery	Demographic factors, maternal factors, obstetric characteristics, pregnancy termination attributes, medical and obstetric history
	Germany	[43]	Comparison between two birth weight groups	Demographic factors, medical and obstetric history, ultrasound measurements
	London	[40]	Predicting labor outcome	Demographic factors, medical and obstetric history, ultrasound measurements
	Timisoara	[53]	Exploring pregnancy factors	Demographic factors, maternal factors, obstetric characteristics, behavioral parameters, current medical record
	Scotland	[52]	Predicting pregnancy risks/complications	Demographic factors, maternal factors, obstetric characteristics, behavioral parameters
	Slovenia	[50]	Predicting pregnancy risks/complications	Demographic factors, maternal factors, obstetric characteristics, EHG related features
South Asia	India	[33, 48, 51, 54]	Predicting pregnancy risks/complications	Demographic factors, maternal factors, obstetric characteristics, medical and obstetric history, behavioral parameters, current medical record
East Asia	China	[39, 41, 45, 55]	Exploring pregnancy factors, predicting mode of delivery and outcome of IVF treatment	Demographic factors, maternal factors, obstetric characteristics, medical and obstetric record, ultrasound measurements, neonatal features, infertility characteristics
	Taiwan	[9]	Exploring pregnancy factors	Demographic factors, maternal factors, obstetric characteristics, behavioral parameters, medical and obstetric history
Middle East	Iran	[34, 46]	Predicting outcome of labor, exploring pregnancy factors	Demographic factors, maternal factors, obstetric characteristics, medical and obstetric history, ultrasound characteristics
	Turkey	[42]	Predicting outcome of IVF treatment	Demographic factors, maternal factors, obstetric characteristics, infertility characteristics
	Israel	[36]	Predicting mode of delivery	Demographic factors, medical and obstetric history, obstetric characteristics, behavioral parameters, neonatal factors
East Africa	Ethiopia	[35, 44]	Exploring pregnancy factors	Demographic factors, obstetric characteristics, maternal factors, medical and obstetric history
Australia	-	[10]	Predicting pregnancy risks/complications	Demographic factors, obstetric characteristics, medical and obstetric history

The studies conducted in *India* (under the region of South Asia) revolve around predicting risk factors in pregnancy, while all the features were related to demographic factors, obstetric characteristics, behavioral parameters, and current medical records. However, the researches conducted in *China* (under the region of East Asia) were related to predicting mode of delivery, for example, predicting vaginal birth after cesarean section [41] and predicting risk factors in emergency cesarean section [39] and other two studies were related to IVF treatment [55], [45]. One study conducted in *Ethiopia* was related to predicting child mortality [44] and other was about identifying factors of successful vaginal delivery [35]. The studies of *Iran* were related to predicting mode of delivery [34] and prediction of low birth weight [46]. Again studies related to the mode of delivery, pregnancy factors, and risk/ complications used the features types of obstetric and demographic characteristics and maternal factors.

The review findings (see Table 5) indicated that the ‘pregnancy risks/ complications’ and ‘mode of delivery’ were focused mostly in nearly all of the regions, while only a few studies focused on the ‘outcome of IVF treatment.’ Furthermore, the research works within this scope are only conducted in *China* and *Turkey,25_hassan2020machine*. As IVF treatment is based on sophisticated technology, developed countries are well suited for this type of research.

## Study findings

This section represents an overview of findings from the detailed systematic review.

### Topical relationship

The word clouds modeled from titles and keywords confirm the strong relationships among the reviewed articles. The highlighted words in the visual representations (Figs. 4, 5) have more prominence in these studies. Significantly highlighted words in both visualizations include data mining, birth, prediction, preterm, delivery, cesarean, factors, and risks. This shows how closely the reviewed papers are related and relevant to each other.

### Publication year and research type

Several articles related to pregnancy have been published since 2000 from those a total of 26 articles were selected for this review study. The ML-related studies focusing on various aspects of pregnancy remarkably increased between 2015 and 2020 with a total of 18 studies. Most of the selected articles (*n* = 20) were journals, some were conference papers (*n* = 6). Most of the selected articles were published in prestigious international journals devoted to medical research, medicine, health informatics, science, and engineering. This demonstrates how the applications of machine learning have advanced dramatically in health care and maternal welfare over time.

### Type of data and features

Though non-identical datasets were used for each study for different motives there was similarity in how the data was collected and the types of features used. The most commonly used feature categories were different demographic and obstetric characteristics, maternal factors; while ultrasound measurement was used in some studies to predict the mode of childbirth, and features related to infertility were used for predicting the outcome of IVF treatment. The review showed that a few distinct features were recurring in almost every study, e.g., age, height, body mass index, parity, and gestational age. For most cases (*n* = 14), researchers collected medical records personally. Again data for a total of 11 studies were collected through cohort studies.

### Study scopes and objectives

The reviewed studies were classified under six scopes. The majority of the studies (*n* = 9, 35%) were concerned about predicting pregnancy risks/ complications. Moreover, under this scope, most studies (*n* = 5, 19%) were conducted to predict preterm birth or extreme preterm birth. Also, some studies (*n* = 2, 8%) were done to explore factors responsible for preterm birth. Some studies were done for predicting mode of delivery (*n* = 4, 15%) and predicting outcomes of IVF treatment (*n* = 3, 11%). Again, studies for predicting pregnancy risks/ complications and predicting outcomes of IVF treatment were published recently.

### ML algorithms

ML has been used mostly in predicting complications in pregnancy, predicting mode of delivery, exploring factors responsible for preterm birth, and predicting vaginal birth-given some prior conditions like the previous cesarean section. To get such outcomes mostly used ML algorithms were SVM, MA, RF, UA, DT, LR, and NB (see Table 3). Also, it was evident that different algorithms gave different accuracy in different scopes. For example, DT was used in predicting birth mode and birth complications which resulted in different accuracy.

### Country context

The reviewed studies were conducted in the following 15 countries: USA, Ethiopia, Iran, India, China, Portugal, Germany, Israel, Scotland, Romania, Australia, Taiwan, Slovenia, Turkey, and England. It was found that almost half of these studies were carried out in the USA (*n*=3), India (*n*=4), and China (*n*=4); while the casualties and occurrences of maternal complications are also noticeable in most of these nations. The scope of studies conducted in different regions was different based on the country context. For example, for the most part, the studies related to IVF treatment-related studies were conducted in China (2 out of 3). While the majority of studies (7 out of 13) related to “predicting pregnancy risks/ complications” and “predicting mode of childbirth” were carried out in Europe (Portugal, Scotland, and Slovenia) and North America (USA).

## Future research implication

The review study revealed multiple open issues for further investigation. Future research can be conducted on the following issues for predicting pregnancy outcomes and ensuring maternal safety.

### Utilizing unsupervised and deep learning

This review study found that almost all the reviewed studies were carried out using supervised ML algorithms (*n* = 23). In the future, some unsupervised ML algorithms such as clustering algorithms can be used to aggregate data in different groups based on mothers’ pregnancy characteristics. These clusters can be carefully analyzed to find out the patterns where maternal complications appear and therefore the cause of these complications can be investigated. In addition, deep learning and ensemble ML can be used in the future since deep learning offers considerable performance improvement in medical diagnosis [61].

### Revealing the unknown reasons of maternal complications

Some maternal complications, for example, preterm birth, infection, preeclampsia, gestational diabetes occur frequently during childbirth [62]. Preterm birth complications are the leading cause of death among children [63]. According to WHO, every year preterm birth accounts for approximately 1 million death [64]. A number of studies (*n* = 7) have been conducted with the goal of predicting preterm birth or determining the factors that cause it. In addition, some major complications like severe bleeding, infection, preeclampsia, and eclampsia are predominantly responsible for maternal deaths [65]. In the future, data associated with these complications and EHR of symptomatic mothers can be collected and then analyzed using effective data mining techniques to discover if there are some unknown reasons behind these complications.

### Developing usable and useful applications

The selected studies related to ensuring maternal safety have been carried out. Though the reviewed studies do not present any software framework, desktop, or mobile application. The data mining model developed [8] was installed in the maternal and perinatal care unit of Centro Hospitalar of Oporto, Portugal for assisting physicians in clinical decision making. In the future, ML-based applications can be developed for predicting maternal complications along with the. In the future, besides conducting research on predicting mode of delivery, maternal complications, etc., ML-based applications can be developed. Consequently, these researches can be utilized in practical applications. Moreover, the usability and usefulness of these software systems can be evaluated to examine how effective they are in aiding doctors in clinical decision-making.

### Enhancing dataset and its accessibility

For most of the studies, the used data sets were not open source. Researchers can effectively contribute to these kinds of studies if the dataset comprising pregnancy characteristics is made open source while hiding personal information. It is a matter of concern that according to WHO, most maternal deaths occur in Sub-Saharan Africa and Southern Asia [66] though very little research has been conducted in these regions. Data can be collected from these regions and some investigation can be carried out to find out the correlation in different features to analyze the reasons for these unfortunate deaths and complications.

### Exploring the potential of surgical robotic tool

Surgical robotic tools have shown increasing effectiveness and efficiency in many surgical operations [67, 68]. With the aid of ML, data from previous successful surgery can be utilized to train this kind of robotic tool. Stable and accurate movements of robotic arms result in precise surgery, reducing the risk of infection and blood loss, shallow surgical incisions, enhanced visions, etc. Future potential research can be carried out to investigate if such robotic tools can be operated to perform cesarean sections.

## Future research framework

**Fig. 8:**
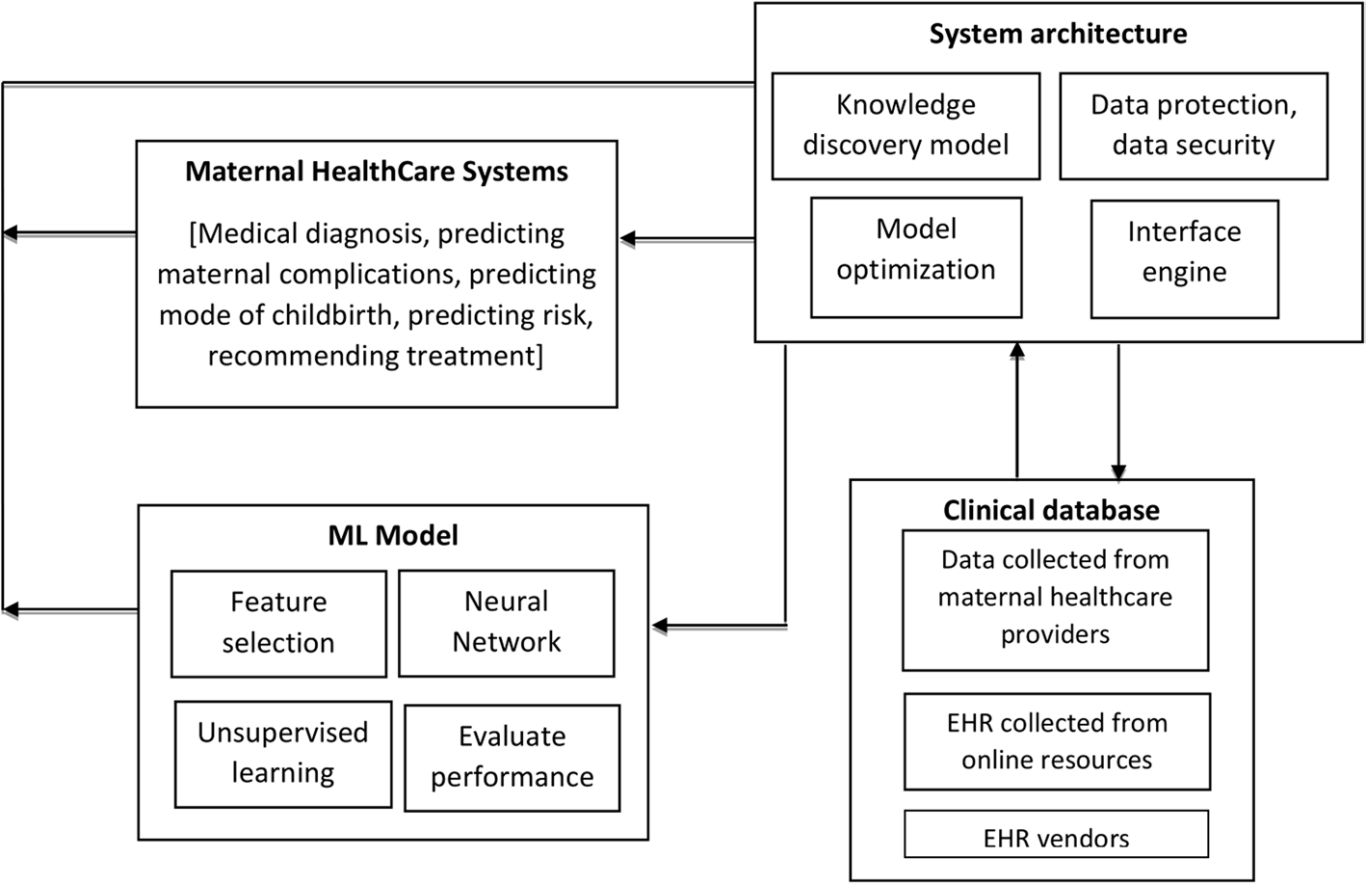
Future research framework for adopting ML in maternal healthcare

The gaps found from this review study led to the proposition of a conceptual framework to advance a machine learning-based healthcare system. The proposed framework based on machine learning for the maternal healthcare system has four interconnected components (see Fig. 8) as discussed below.

(i) **Clinical Database:** The clinical database serves as the foundation for the predictive model as decisions are made depending on the patients’ information. The database stores pregnancy characteristics of mothers in a structured manner concealing personal information. Data reliability can also be improved by encrypting data and ensuring data backup on another system. The information may be gathered from health services providers, EHR vendors, and reliable online sources. Since these data will be used to make medical decisions, they must be authentic and accurate. As a result, prospective researchers must prioritize the management of the clinical database to assure its trustworthiness.
(ii) **System Architecture:** The knowledge discovery model (KDM) is a key component of the system architecture. KDM is responsible for identifying and discovering meaningful patterns and correlations in EHR, evaluating the performance of the ML model, and presenting known knowledge in an organized way. As ML is progressing at a frenetic rate, the machine learning-based architecture will also likely undergo some changes with the growth of ML. Hence the system will be optimized and improved by a model optimization module. Interface engine processes data and manages interconnection among different components of the system. As a security measure, the data protection and data security component will concentrate on the systems’ management and ensure that the system’s security is not jeopardized.
(iii) **ML Model:** The central component of the framework is the machine learning model, as this model can assist physicians in determining the pregnancy’s outcome. To create this model, features must be efficiently chosen, machine learning algorithms must be incorporated, and accuracy must be evaluated. As a result, potential researchers must concentrate on identifying the critical features that have the greatest impact on the outcome, as well as selecting the appropriate algorithms and ensuring that the model is accurate.
(iv) **Maternal Health Care System:** With the evolution of machine learning and data mining in health sectors, researchers should concentrate on how to use ML in maternal sectors. Through this review, it is evident that machine learning techniques can be used in medical diagnosis, predicting maternal complications, predicting mode of childbirth, and predicting risk. Therefore future researchers should focus on using machine learning on maternal records so that it can help the professionals with diagnosis, with detecting different maternal complications or risks, and help them with prescribing treatments to the patients accordingly.

## Conclusion

In this review, the existing articles were investigated systematically, to explore the state of the art views, find out the future research scopes, and the limitations of the existing studies focusing on the use of ML and predicting pregnancy outcomes. To obtain the first research objective (RO1), this article summarizes and synthesizes the studies carried out in the past years focusing on pregnancy outcomes using ML and depicting the publication profile of the reviewed studies. To address RO2, data sources and features used in the reviewed articles were explored. This systematic review showed the diverse objectives of the reviewed articles which may motivate the researchers to set a new objective for future research. The review also analyzed the algorithms used in different articles to obtain RO3, which will facilitate to gain a basic understanding of what kind of algorithms can be used in the focused area; what type of features need to be considered to achieve a specific objective; which algorithm is better suited for the type of data collected; and the like. To obtain RO4, some gaps in existing literature were identified, and to meet these gaps some potential research opportunities were also suggested. In addition, based on the findings, this review study proposed a framework adopting the ML algorithms for the future advancement of maternal healthcare through monitoring the progress of pregnancy; diagnosing avoidable pregnancy complications; recommending treatment to patients, and aiding the clinicians in decision making. The revealed research gaps, proposed future research directions, and the proposed framework will foster pursuing the future research.

There are some shortcomings to this review article that must be acknowledged. The keywords used for searching may not encompass all the relevant articles. Again, the inclusion criteria used for selecting the articles may not be able to cover all the related articles. Finally, some papers may be excluded due to the chosen inclusion-exclusion criteria. Since the selection and applying the inclusion-exclusion criteria is a subjective manner that mostly depends on the analyzer.

This review paper will help scholars and practitioners (health professionals) to understand the significance of ML in real-time decision-making regarding pregnancy and can be a prominent aid in reducing maternal threats.

## Supplementary Information

**Additional file 1**.

## Data Availability

The datasets generated and analyzed during the current study are not publicly available due to the fact that we are still using it for another publication, but are available from the corresponding author on reasonable request.
