# Supplementary material for: Machine learning to predict pregnancy outcomes: a systematic review, synthesizing framework and future research agenda
Source: BMC Pregnancy Childbirth. 2022 Apr 22;22:348. doi: 10.1186/s12884-022-04594-2 (PMC9097057; doi:10.1186/s12884-022-04594-2)
Supplement: Supplementary file 1 — Additional file 1. [file 12884_2022_4594_MOESM1_ESM.pdf]

APPENDIX

Table A1: Types of data and features used

Data Collection Approach	Sample Size	Type of Features	Feature Examples	Ref
Independently collected	4,236	Demographic factors, maternal factors, obstetric characteristics	weight, height, body mass index (BMI), number of gestation weeks	[8]
	600	Demographic factors, maternal factors, obstetric characteristics, medical and obstetric history	age, height, weight, BMI, parity, gestational diabetes	[33]
	1,124	Demographic factors, obstetric characteristics, pregnancy termination attributes, medical and obstetric history	age, parity, gestation week, failed pregnancy termination	[47]
	189	Demographic factors, obstetric characteristics behavioral parameters, current medical record, medical and obstetric history	age, hypertension, history of previous preterm delivery, presence of uterine irritability	[54]
	150	Demographic factors, maternal factors, obstetric characteristics, current medical record	age, BMI, type of birth, gestational age	[48]
	450	Demographic factors, maternal factors, obstetric characteristics, medical and obstetric history	age, height, BMI, number of fetuses, number of pregnancies, history of abortion	[46]
	25,689	Demographic factors, obstetric characteristics	age, gestational age, ethnicity	[49]
	546	Demographic factors, maternal factors, obstetric characteristics, behavioral parameters, current medical record	gastrointestinal disease, BMI, previous birth, weight	[53]
	1,729	Demographic factors, infertility characteristics	age, infertility diagnosis, infertility factor, number of cleavages	[42]
	31,030	Maternal factors, obstetric characteristics, infertility characteristics	BMI, duration of infertility, embryo transferred count, embryo grade	[45]
	11,855	Demographic factors, maternal factors, obstetric characteristics, medical and obstetric history	gestational diabetes, previous vaginal delivery, ethnicity, BMI, estimated gestational age	[37]
Prospective cohort study	910	Demographic factors, maternal factors obstetric characteristics, behavioral parameters, medical and obstetric history	age, hypertension, parity, previous preterm birth	[9]
	1,743	Demographic factors, medical and obstetric history, ultrasound measurements	parity, cervical length, gestational age at delivery, ethnicity	[40]
	100	Demographic factors, maternal factors, ultrasound characteristics	age, Bishop score, initial weight, initial BMI	[34]
	1,054	Maternal factors, demographic factors, obstetric characteristics	age, BMI, parity, duration of pregnancy, gestational diabetes	[43]

Retrospective cohort study	9,888	Demographic factors, medical and obstetric history, obstetric characteristics, behavioral parameters, neonatal factors	age, parity, gestational age, head circumferences	[36]
	599	Demographic factors, maternal factors, obstetric characteristics, medical and obstetric history, neonatal features	age, BMI, prior vaginal delivery, gravity, parity, complications of pregnancy	[38]
	10,295	Demographic factors, maternal features, medical and obstetric history, neonatal features, obstetric characteristics	age, BMI, birth weight, gravidity, parity, previous cesarean	[39]
	2,006	Demographic factors, maternal factors, obstetric characteristics, medical and obstetric record, ultrasound measurements	gestational age, parity, history of abortion, history of vaginal delivery, estimated birth weight	[41]
	7,188	Demographic factors, maternal factors, obstetric characteristics, medical and obstetric history, infertility characteristics	previous miscarriage, previous abortion, BMI, duration of infertility	[55]
	274	Demographic factors, obstetric characteristics, medical and obstetric history	miscarriages, previous preterm birth, multiple gestation, cervical length	[10]
	313,238	Demographic factors, maternal factors, obstetric characteristics, behavioral parameters	age, height, smoking status, Apgar score, birth weight	[52]
Case-control study	204	Demographic factors, maternal factors, medical and obstetric history obstetric characteristics	age, gestational age, inter delivery interval, prior successful vaginal birth after cesarean section	[35]

Table A2: Categories of the features used in reviewed articles

Categories	Feature Example
Demographic factors	age, education, ethnicity, occupation, religion, marital status
Maternal factors	height, weight, BMI, weight on conceiving, BMI at delivery, BMI before pregnancy
Obstetric characteristics	mode of delivery, sex of the baby, birth weight, bishop score, gestational age, hypertension, gastrointestinal disease, gestational diabetes, induction of labor, duration of pregnancy, uterine rupture, complications of pregnancy, complications of labor and delivery, induction of labor, low lying placenta, cervical dilatation, presence of meconium
Medical and obstetric history	pregnancy parity, gravidity, history of gestational diabetes, history of abortion, history of previous preterm delivery, previous vaginal delivery, previous preterm vaginal delivery, vaginal delivery after previous cesarean delivery, maximum birth weight of previous child, minimal gestational week in previous deliveries, previous ectopic pregnancies, prior uterine layer closure, previous cesarean, previous miscarriage
Medical history of relatives	family history of preeclampsia, family history of gestational diabetes
Current medical record	blood pressure, weight gain, number of physician visits, glucose level, allergies, medicines taken during pregnancy, blood type
Pregnancy termination attributes	achieved pregnancy termination, failed pregnancy termination, incomplete pregnancy termination, contraceptive method
Behavioral parameters	smoking, drugs, alcohol

Ultrasound measurements	cervical length, fetal head-pubis symphysis distance, biparietal diameter, abdominal circumference, femur length, humerus length, head circumference
Neonatal features	neonatal mortality, 5-minute apgar test, post-partum hemorrhage, uterine rupture, macrosomia
Infertility characteristics	type of infertility, duration of infertility, infertility diagnosis, infertility factor, antral follicle counts, number of cleavages, frozen sperm, thyroid stimulating hormone, embryo transferred count, embryo Grade

Table A3: Performance of the machine learning algorithms

Algorithm	Reference	Performance
Decision Tree	[8]	Accuracy = 83.91%, Specificity = 80.05%, Sensitivity = 88.28%
	[47]	Sensitivity = 92.6%
	[54]	Accuracy = 89.95%, Sensitivity = 97.69%, Specificity = 72.88%
Naive Bayes	[8]	Accuracy = 74.69%, Specificity = 62.98%, Sensitivity = 84.29%
	[54]	Accuracy = 77.78%, Sensitivity = 90.00%, Specificity = 50.85%
Generalized Linear Models	[8]	Accuracy = 77.41%, Specificity = 67.55%, Sensitivity = 84.27%
	[47]	Sensitivity = 92.9%
Support Vector Machine	[8]	Accuracy = 62.06%, Specificity = 27.92%, Sensitivity = 85.57%
	[47]	Sensitivity = 92.9%
	[54]	Accuracy = 74.07%, Sensitivity = 98.46%, Specificity = 20.34%
	[42]	Accuracy = 98.01%, F-score = 98.1%, AUC = 99.3%
	[51]	Accuracy = 86.11%, Sensitivity = 86.11%, Precision = 86.11%
	[50]	Precision = 49.13%, Sensitivity= 46.14%, Specificity= 94.09%, AUC= 63.2%
Gradient Boosting	[36]	AUC = 79.3%
Random Forest	[36]	AUC = 75.6%
	[54]	Accuracy= 70.90%, Sensitivity = 99.23%, Specificity = 8.47%
	[10]	Accuracy= 95%, Sensitivity= 100%, Specificity= 94%, AUC= 98%
	[42]	Accuracy = 98.83%, F-score = 98.8%, AUC = 99.2%
	[50]	Precision = 95.86%, Sensitivity = 98.4%, Specificity = 99.76%, AUC = 99.9%
Balanced Random Forest	[36]	AUC = 78.2 %
AdaBoost Ensemble	[36]	AUC = 78.4 %
C4.5	[33]	Accuracy = 71.30%

	[42]	Accuracy = 93.21%, F-score = 93.2%, AUC = 96.6%
Multivariate Analysis	[38]	AUC = 72.3 %
Logistic Regression	[69]	AUC = 66 %
	[54]	Accuracy = 74.07%, Sensitivity = 92.31%, Specificity = 33.90%
	[51]	Accuracy = 87.23%, Sensitivity = 98.75%, Specificity = 21.43%, Precision = 98.75%
Neural Network	[69]	AUC = 64%-68%
	[54]	Accuracy= 76.19%, Sensitivity= 93.85%, Specificity= 37.29%
	[49]	AUC = 82.7%, Sensitivity = 96.5%
	[10]	Accuracy= 92%, Sensitivity= 50%, Specificity= 100%, AUC= 76%
J48	[44]	Accuracy=94.3%, Sensitivity=93.8%, Specificity=94.3%, Area under ROC =94.8%
	[10]	Accuracy= 81%, Sensitivity= 33%, Specificity= 90%, AUC= 63%
PART	[44]	Accuracy = 84.3%
XGBoost	[55]	AUC = 73%
K Nearest Neighbor	[10]	Accuracy = 95%, Sensitivity = 67%, Specificity = 100%, AUC = 98%
	[50]	Precision = 34.91%, Sensitivity= 92.68%, Specificity= 99.28%, AUC= 71.7%
Multilayer Perceptron Neural Networks	[42]	Accuracy = 97.77%, F-score = 97.8%, AUC = 99.1%
Classification and Regression Trees	[42]	Accuracy = 95.24%, F-score = 95.2%, AUC = 97%
